# Inhibition of MAPK-mediated ACE expression by compound C66 prevents STZ-induced diabetic nephropathy

**DOI:** 10.1111/jcmm.12175

**Published:** 2013-12-11

**Authors:** Yong Pan, Yi Huang, Zhe Wang, Qilu Fang, Yusheng Sun, Chao Tong, Kesong Peng, Yangwei Wang, Lining Miao, Lu Cai, Yunjie Zhao, Guang Liang

**Affiliations:** a Chemical Biology Research Center, School of Pharmaceutical Sciences, Wenzhou Medical UniversityWenzhou, Zhejiang, China; b Chinese-American Research Institute for Diabetic Complications, School of Pharmaceutical Sciences, Wenzhou Medical UniversityWenzhou, Zhejiang, China; c Department of General Surgery, The First Affiliated Hospital of Wenzhou Medical UniversityWenzhou, Zhejiang, China; d Department of Nephropathy, the Second Hospital of Jilin UniversityChangchun, Jilin, China

**Keywords:** renin-angiotensin system, diabetic nephropathy, angiotensin converting enzyme, mitogen-activated protein kinases, (2E,6E)-2,6-bis(2-(trifluoromethyl)benzylidene)cyclohexanone

## Abstract

A range of *in vitro*, experimental and clinical intervention studies have implicated an important role for hyperglycaemia-induced activation of the renin-angiotensin system (RAS) in the development and progression of diabetic nephropathy (DN). Blockade of RAS by angiotensin converting enzyme (ACE) inhibitors is an effective strategy in treating diabetic kidney diseases. However, few studies demonstrate the mechanism by which hyperglycaemia up-regulates the expression of ACE gene. Our previous studies have identified a novel curcumin analogue, (2E,6E)-2,6-bis(2-(trifluoromethyl)benzylidene)cyclohexanone (C66), which could inhibit the high glucose (HG)-induced phosphorylation of mitogen-activated protein kinases in mouse macrophages. In this study, we found that the renal protection of C66 in diabetic mice was associated with mitogen-activated protein kinase (MAPK) inactivation and ACE/angiotensin II (Ang II) down-regulation. Generally, MAPKs have been considered as a downstream signalling of Ang II and a mediator for Ang II-induced pathophysiological actions. However, using C66 and specific inhibitors as small molecule probes, *in vitro* experiments demonstrate that the MAPK signalling pathway regulates ACE expression under HG stimulation, which contributes to renal Ang II activation and the development of DN. This study indicates that C66 is a potential candidate of DN therapeutic agents, and more importantly, that reduction in ACE expression by MAPKs inhibition seems to be an alternative strategy for the treatment of DN.

## Introduction

Diabetic nephropathy (DN) has become the main cause of morbidity and mortality in the diabetic population. It appears to be multifactorial in origin, involving a number of key pathways, including advanced glycation, activation of intracellular signalling molecules such as protein kinase C, and increased generation of reactive oxygen species 1. The renin-angiotensin system (RAS) plays a crucial role in the regulation of renal and cardiovascular function and has been considered to be involved in the pathological processes that result in DN 2. It has been found that hyperglycaemia in diabetes causes activation of systemic and/or the local RAS in the kidney. Incubation in cultural medium with a high concentration of glucose also induces the overexpression of both angiotensin converting enzyme (ACE) and angiotensin II receptor (AT) 1 in a variety of cell lines including renal cells 3–5. As an outcome of RAS activation, angiotensin II (Ang II) can promote the development of DN by causing hypertrophy of various renal cells, increasing renal microvascular pressure, and inducing inflammation, apoptosis, reactive oxygen species, and podocyte autophagy 1,5. Evidence from both animal and clinical studies supports the hypothesis that blockade of RAS by ACE inhibitors (ACEIs) and angiotensin receptor blockers (ARBs) is an effective strategy in treating diabetic kidney diseases 6.

The mitogen-activated protein kinase (MAPK) cascade is an important intracellular mediator of responses related to cell growth and differentiation, stress, survival and cell death 7. It constitutes three kinase networks, including extracellular regulated kinase (ERK), c-Jun NH_2_-terminal kinase (JNK) and p38 MAPK, all of which have been reported to be activated in response to inflammatory and stressful stimuli, including diabetic conditions or high glucose levels 8,9. Exposure to high glucose (HG) activated MAPKs in rat mesangial cells and tubular epithelial cells 8,10. Previous studies in animals and humans demonstrated that the activation of MAPKs was involved in DN and contributed to the development of DN 9,11–13.

It is generally accepted that MAPKs could be activated by Ang II stimulation and mediate a series of pathological and cellular responses induced by Ang II in diabetic kidney or renal cells 14,15. On the other hand, some studies showed that the activation of MAPKs might regulate ACE gene expression and then activate RAS, including oxidized low-density lipoprotein-16 and bleomycin-induced 12 ERK activation, ramiprilat-induced JNK activation 17, and asymmetric dimethylarginine-triggered phosphorylation of p38 18. However, the current understanding of the relationship between MAPKs and RAS in the pathophysiology of diabetic complications is still limited. It is unclear whether MAPKs mediate HG or hyperglycaemia-induced RAS activation and regulate the overexpression of RAS-related genes in DN.

Curcumin is the main active component of the natural turmeric and has been extensively demonstrated as a multifunctional agent on cancer, inflammation and inflammatory-related diseases. However, the clinical application of curcumin has been significantly limited by its poor bioavailability 19. Design of synthetic structural analogues of curcumin is one approach to obtain better small molecule than the lead. Our group has been engaged in curcumin structural modification for a long time. Previously, we have synthesized and evaluated a series of curcumin mono-carbonyl analogues with improved pharmacokinetic profiles *in vivo* 19. As an outcome of our previous studies, a novel curcumin analogue, (2E,6E)-2,6-bis(2-(trifluoromethyl)benzylidene)cyclohexanone (C66), was identified as a new candidate, which could inhibit the HG-induced MAPK phosphorylation and downstream inflammatory response in mouse macrophages 19. In this study, we found that the renal protection conferred by C66 in diabetic mice (DM) was accompanied by MAPK inactivation and ACE/Ang II down-regulation. Furthermore, using C66 and specific inhibitors as small molecule probes, our *in vitro* experiments demonstrated the possibility that the MAPK signalling pathway regulates ACE expression and RAS activation under HG stimulation.

## Materials and methods

### Reagents

**Fig 1:**
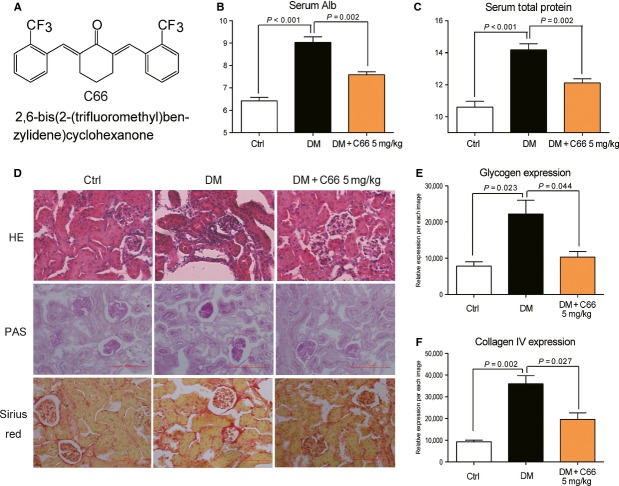
C66 administration significantly affected metabolic profiles and improved renal histological abnormalities of diabetic mice. (A) Chemical structure of C66. (B–C) Increased serum albumin and serum total protein levels in diabetic mice were reversed by C66 treatment, respectively. (D) Representative figures of histological abnormalities in diabetic renal tissues (200×). Haematoxylin and eosin staining was used for analysis of histological abnormalities; periodic acid and schiff and sirius red stainings were used for the detection of glycogen (purple) and type IV collagen (red) in kidney section. (E–F) The relative density of glycogen (E) and collagen IV (F) expression per image were counted in five vision fields of 100-μm length across the kidney. Data are presented as mean ± SEM, seven mice in each group (DM = diabetic mice).

Glucose, mannitol and streptozotocin (STZ) were purchased from Sigma-Aldrich (St. Louis, MO, USA). Compound C66 was synthesized and characterized in our previous publication 20. Before use in the biological experiments, C66 was re-crystallized from CHCl_3_/EtOH, and then a high-performance liquid chromatography method was used to determine its purity (98.36%). The structure of C66 is shown in Figure 1A. C66 was dissolved in dimethyl sulfoxide (DMSO) for *in vitro* experiments and was dissolved in 1% sodium carboxyl methyl cellulose (CMC-Na) for *in vivo* experiments. Anti-pERK, anti-pp38, anti-pJNK, anti-JNK and anti-transforming growth factor-β1 (TGF-β1) antibodies were purchased from Cell Signalling Technology (Danvers, MA, USA). Anti-ACE, anti-ERK, anti-p38 and anti-GAPDH antibodies were purchased from Santa Cruz (San Diego, CA, USA).

### Cell culture

A rat renal tubular epithelial cell line (NRK-52E) was obtained from the Institute of Biochemistry and Cell Biology, CAS (Shanghai, China) and cultured in DMEM medium (Gibco, Eggenstein, Germany) containing 5.5 mmol/l D-glucose (low glucose, LG) supplemented with 10% FBS (Hyclone), 100 U/ml penicillin and 100 mg/ml streptomycin. Cells were grown in an atmosphere of 5% CO_2_ in a humidified incubator. Before treatment, NRK-52E cells were cultured in 60-mm plates for overnight.

### Animal experiments

Protocols for animal studies were approved by the Wenzhou Medical College Animal Policy and Welfare Committee (Approved documents: 2009/APWC/0031). Male C57BL/6 mice, weighing 18–22 g at 8 weeks of age, were obtained from the Animal Center of Wenzhou Medical College (Wenzhou, China). Animals were housed at 22°C with a 12:12 hrs light/dark cycle and water and a standard mouse diet were consumed. To induce type 1 diabetes, mice were treated with a single intraperitoneal injection of STZ (150 mg/kg in citrate buffer, pH = 4.5), while the control animals were received the same volume of citrate buffer. The blood glucose level was monitored on days 3 and 7 after the STZ injection using a glucometer. Seven days after STZ injection, mice with fasting-blood glucose >12 mmol/l were considered diabetic, and then randomly divided into two groups: DM (*n* = 7), and C66-treated DM (DM+C66, *n* = 7). In the DM+C66 group, mice were orally administrated with C66 at 5 mg/kg once every 2 days. The DM group and age-matched control group (*n* = 7) received 1% CMC-Na solution alone according to the same schedule. Bodyweight and blood glucose were recorded on days 7, 17, 27, 47, 57 and 67 after C66 administration. On day 67, animals were killed under ether anaesthesia. Kidney tissues were embedded in 4% paraformaldehyde for pathological analysis and/or snap-frozen in liquid nitrogen for gene and protein expression analysis. The blood was also collected at the time of death.

### Cellular immunofluorescence assay of ACE

Cells were fixed in 4% paraformaldehyde, then treated with 5% H_2_O_2_ for 10 min. and with 3% BSA for 30 min. Slides were incubated overnight at 4°C with anti-ACE antibody, then incubated with PE-labelled goat antimouse IgG for 1 hrs at room temperature. The nucleus was then stained with DAPI for 5 min., and the images were viewed with a fluorescence microscope (200×; Nikon, Tokyo, Japan).

### Determination of serum Ang II

The level of Ang II in mouse serum was measured with an Ang II ELISA kit (R&D Systems, Minneapolis, MN, USA) according to the manufacturer's instructions.

### Determination of serum creatinine, serum albumin and serum total protein

The levels of creatinine, albumin (ALB) and total protein in mouse serum were determined by an automatic biochemical analyzer (Olympus AU5400; Olympus Corporation, Tokyo, Japan) according to the manufacturer's instructions.

### Real-time quantitative PCR

Total RNA was isolated from tissues and cells using TRIZOL (Invitrogen, Carlsbad, CA, USA). Reverse transcription and quantitative PCR were performed with an M-MLV Platinum RT-qPCR Kit (Invitrogen). Real-time quantitative PCR was carried out using the Eppendorf Realplex^4^ instrument (Eppendorf, Hamburg, Germany). The primer sequences of genes are shown in Table S1. The relative amount of each gene was normalized to that of GAPDH gene.

### Western blotting

After lysate homogenates of tissues or cells were prepared, Western blotting were performed as previously described unless otherwise indicated 19. All cellular Western blots were repeated at least three times. The density of the immunoreactive bands was analysed using Image J software (NIH, Bethesda, MD, USA).

### Histopathology

Kidneys were fixed in 4% paraformaldehyde solution, embedded in paraffin and sectioned at 5 μm. After dehydration, sections were stained with haematoxylin and eosin. To evaluate the histopathological damage, each image of sections was examined by a light microscope (200× amplification; Nikon).

### Periodic acid and schiff staining for glycogen and sirius red staining for type IV collagen

Renal tissues were fixed in paraformaldehyde solution and embedded in paraffin. Paraffin sections (5 μm) of the tissues were stained with 0.5% periodic acid and schiff solution (PAS) to evaluate glycogen distribution, or were stained with 0.1% Sirius Red F3B and 1.3% saturated aqueous solution of picric acid to evaluate type IV collagen presence. The stained sections were then viewed with a light microscope (200× amplification; Nikon).

### Statistical analysis

Data were collected from at least three independent experiments for *in vitro* studies and seven mice in each group for the *in vivo* studies, and were presented as mean ± SD. anova and GraphPad Pro (GraphPad, San Diego, CA, USA) were used to analyse the statistical significance between sets of data. Differences were considered to be significant at *P* < 0.05.

## Results

### Administration of C66 attenuated the metabolic and histological abnormalities of diabetic kidneys

We previously found that C66 was beneficial for the prevention of DN in rats 19. In this study, a mouse model of STZ-induced type 1 diabetes was used. Seven days after STZ injection, mice developed overt diabetes (blood glucose >20 mM). The mice in DM+C66 group were orally treated with C66 at 5 mg/kg every other day for 2 months. Figure S1A shows that there was no significant difference in blood glucose profile between the DM control group and the DM+C66 group, indicating that C66 treatment did not affect blood glucose level in the type 1 DM. However, the serum biochemical analysis showed that C66 treatment for 2 months significantly decreased the levels of the serum creatinine (Fig. S1B), serum ALB (Alb, Fig. 1B) and serum total protein (STP; Fig. 1C) induced by hyperglycaemia (*P* < 0.001), indicating that C66 administration attenuated the indexes of DN in mice. Staining with haematoxylin and eosin, PAS and Sirius Red further validated the histological renal improvement in C66-treated DM when compared to that of the DM group. As shown in Figure 1D, renal structural disorder, inflammatory macrophage infiltration, and increased glycogen and collagen IV were observed in the diabetic kidney (DM) group. Fibrosis was particularly severe in the glomerulus and tubular area, where the majority of infiltrating immunocytes were found (haematoxylin and eosin staining in Fig. 1D). However, these pathological changes were significantly attenuated in DM+C66 group. The quantitative counting of glycogen and collagen IV were carried out and the results indicated that inhibition through treatment with C66 is of statistical significance (Fig. 1E and F).

### Effect of C66 administration on renal MAPK and RAS in type 1 DM

**Fig 2:**
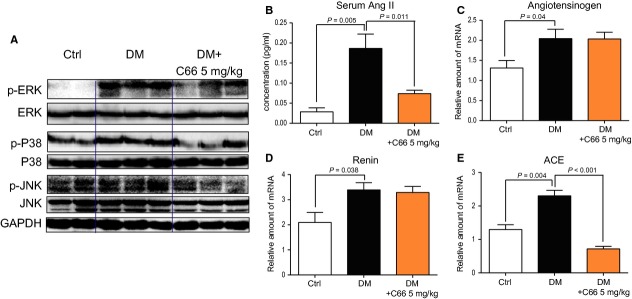
C66 treatment affected renal mitogen-activated protein kinase (MAPK) phosphorylation and renin-angiotensin system activation in diabetic mouse kidneys. (A) Western blot analysis of extracted proteins from the representative mouse kidney tissues showing that the diabetes-induced phosphorylation of MAPKs (extracellular regulated kinase, JNK, and p38) was reversed by C66 treatment. (B) ELISA analysis of serum showing that diabetes-increased serum Ang II level was prevented by C66 treatment (*n* = 7/group). (C–D) Total RNA extraction in mouse kidney tissues was performed as described in Materials and Methods. The mRNA expression levels of these three genes detected by RT-qPCR. RNA levels (mean ± SEM) are expressed as a ratio of GAPDH gene (*n* = 7/group).

We determined the effects of C66 administration on hyperglycaemia-induced MAPK activity in diabetic kidneys. Figure 2A shows that, compared with control group, DM had increased phosphorylation of MAPKs in kidney tissues, while C66 administration significantly inhibited diabetes-induced ERK1/2, p38 and JNK phosphorylation. These results are consistent with our previous experiments in rats 19. To assess the possible effects of C66 on RAS and the possible association between MAPKs and RAS, we determined the levels of serum Ang II and RAS-related gene expression in the kidney tissues of the three groups. As shown in Figure 2B, the serum Ang II in DM has increased 7.4-fold compared to the control mice, indicating that RAS was activated in the diabetic condition, while administration with C66 for 2 months significantly decreased the serum Ang II level. In a classic RAS pathway, juxtaglomerular cells in the kidneys secrete renin, which carries out the conversion of angiotensinogen to angiotensin I (Ang I), and Ang I is subsequently converted to Ang II by the ACE 2. Thus, we measured the gene expression of angiotensinogen, renin, and ACE in the kidneys of three animal groups (Fig. 2C–E). These three genes are unsurprisingly increased in diabetic kidneys. Interestingly, C66 administration does not affect angiotensinogen and renin expression, but significantly reduced ACE mRNA levels (*P* < 0.001, Fig. 2E), indicating that the reduction in Ang II level by C66 may result from its inhibition of hyperglycaemia-induced ACE overexpression. Thus, these results provide us with a possible association between MAPKs and RAS, and in particular between MAPKs and ACE expression in DN.

### HG incubation increased ACE and TGF-β1 expression and MAPK phosphorylation in NRK-52E cells

**Fig 3:**
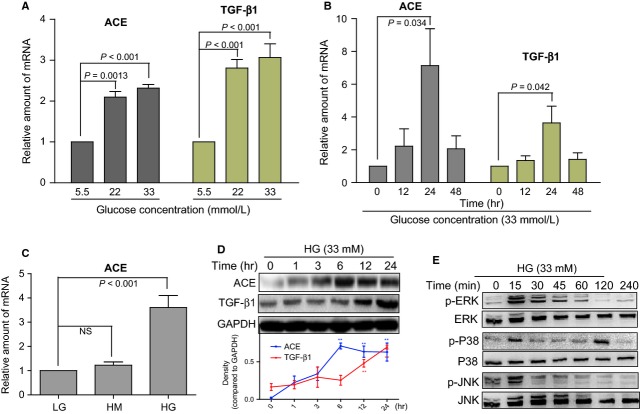
Effects of high glucose (HG) on angiotensin converting enzyme (ACE), transforming growth factor-β1 (TGF-β1), and mitogen-activated protein kinase (MAPK) in NRK-52E cells. Cells were seeded in 60-mm plates with each well containing 2.5 × 10^6^ cells, then treated with different glucose concentrations (5.5, 22 or 33 mM) for several sequential intervals. After treatment, total mRNA or proteins were extracted, and they were analysed by RT-qPCR (normalized to GAPDH gene) or Western blot, respectively. (A) High glucose dose-dependently increased the mRNA expression of ACE and TGF-β1. (B) The time-course effects of 33 mM HG on the mRNA levels of ACE and TGF-β1. (C) Incubation with high mannitol at 33 mM for 24 hrs did not induce ACE mRNA overexpression. (D) The time-course effects of 33 mM HG on the protein levels of ACE and TGF-β1 with GAPDH as a loading control. The curve figures show the normalized optical density from the data more than four independent experiments. (F) The time-course effects of 33 mM HG on the phosphorylation of MAPKs with the respective total MAPK proteins as loading controls. Results are representative of four independent experiments.

In order to determine the relationship between fibrosis, MAPKs activation and ACE expression in the HG-stimulated renal cell model, renal tubular epithelial cells (NRK-52E) were treated with glucose at different concentrations for the indicated time. Relative mRNA expressions of ACE and TGF-β1, a marker of fibrosis, were measured with real-time RT-qPCR. Figure 3A shows that D-glucose treatment for 24 hrs dose-dependently (5.5, 22, and 33 mM) increased the mRNA expression of ACE and TGF-β1 more than twofold over LG (5.5 mM) control values. Figure 3B shows the time course of HG (33 mM)-induced ACE and TGF-β1 mRNA expression, which reached a peak at 24 hrs after HG treatment, and was reduced after the 24 hrs incubation possibly because of the absorption and degradation of glucose. To examine whether high osmotic pressure plays a role in HG-induced increase in ACE expression, cells were treated with 5.5 mM D-glucose plus 27.5 mM mannitol (HM) for 24 hrs and expression of the ACE gene was measured. The result in Figure 3C shows that treatment with mannitol did not change the ACE profile. Furthermore, we determined the protein expression of ACE and TGF-β1 in HG-treated cells. Figure 3D shows that HG treatment elicited sustained overexpression of ACE protein for 3–24 hrs as well as TGF-β1 protein for 12–24 hrs. We also detected the time course of HG-induced MAPK phosphorylation in NRK-52E cells. However, the time course of these effects in MAPKs was significantly different from those in ACE and TGF-β1. The phosphorylation of ERK, p38 and JNK reached a peak after a short time (15 min.), and then reduced to a normal level in 1 hr (Fig. 3E). Thus, it is observed that HG-induced MAPK activation occurred first (15 min.) and then ACE/TGF-β1 expression occurred, indicating the possibility that HG-induced ACE expression is mediated by MAPK activation.

### C66 inhibited HG-induced expression of ACE and TGF-β1 in NRK-52E cells

**Fig 4:**
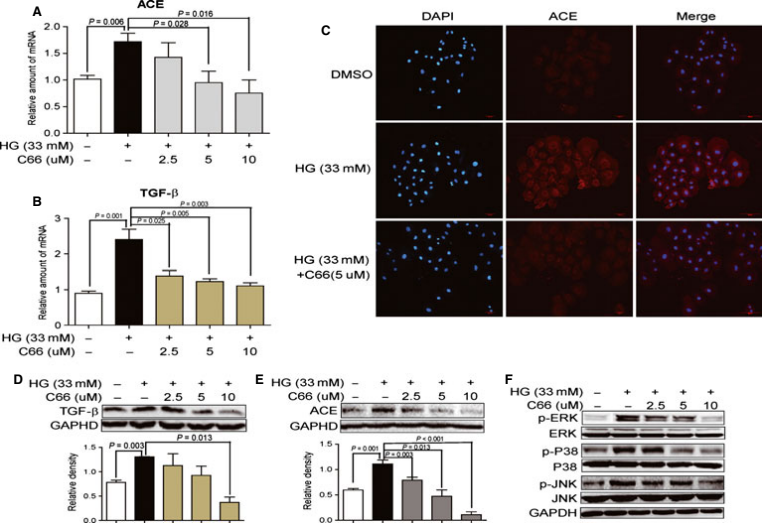
C66 inhibited high glucose (HG)-induced expression of angiotensin converting enzyme (ACE) and transforming growth factor-β1 (TGF-β1), and phosphorylation of mitogen-activated protein kinase (MAPKs) in NRK-52E cells. Cells were pre-treated with C66 (2.5, 5 and 10 μM) or DMSO for 2 hrs, then stimulated with HG at 33 mM for indicated times. After treatment, total mRNA or proteins were extracted, and they were analysed by RT-qPCR (normalized to GAPDH gene), immunostaining, or Western blot, respectively. (A and B) C66 dose-dependently reduced HG-induced mRNA expression of ACE (A) and TGF-β1 (B). (C) Immunofluorescence detection indicated C66 treatment reduced HG-increased ACE protein expression. Nuclei are stained with DAPI (blue), and ACE protein is stained with anti-ACE antibody (red). (D–E) C66 dose-dependently reduced HG-induced protein expression of ACE (D) and TGF-β1 (E). Results are representative of four independent experiments. (F) C66 dose-dependently inhibited HG-induced activation of extracellular regulated kinase, p38 and JNK. Total MAPK proteins and GAPDH were used as loading controls.

We then tested the effects of C66 on ACE/TGF-β1 expression and MAPK phosphorylation in HG-stimulated NRK-52E cells. As shown in Figure 4A and B, C66 dose-dependently reduced the gene expression of both ACE and TGF-β1 induced by 33 mM HG for 24 hrs. Similar results were also observed with immunofluorescence staining (Fig. 4C) and Western blotting (Fig. 4D and E) in NRK-52E cells exposed to 33 mM HG for 3–24 hrs. The inhibitory effects of C66 on MAPK activation were also determined. As shown in Figure 4F, HG incubation for 15 min. increased MAPK phosphorylation, while pre-treatment with C66 dose-dependently inhibited HG-induced phosphorylation of ERK, p38 and JNK. These data demonstrate that C66 could significantly inhibit HG-induced ACE and TGF-β1 expression at the levels of both mRNA and protein, and indicate that the attenuation of HG-caused RAS activation and fibrosis by C66 may be associated with its inhibition on the MAPK signalling pathway.

### MAPK-specific inhibitors inhibited ACE/TGF-β1 but did not affect renin expression in HG-induced NRK-52E cells

**Fig 5:**
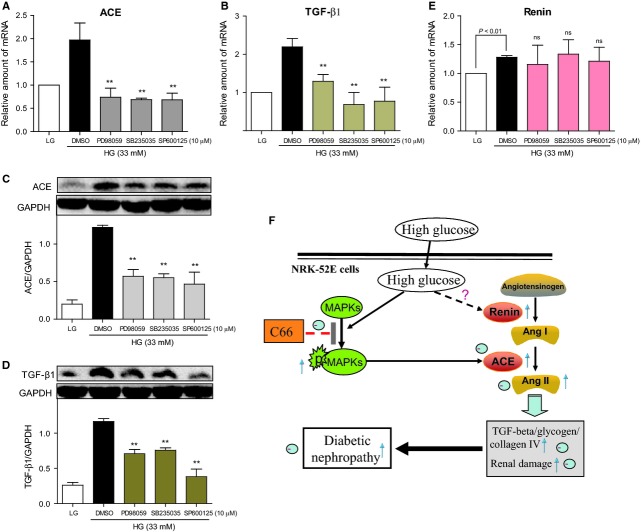
Mitogen-activated protein kinase (MAPKs) are involved in high glucose (HG)-induced diabetic nephropathy signalling cascades. (A–E) NRK-52E cells were pre-treated with PD98059 (extracellular regulated kinase inhibitor), SB235035 (p38 inhibitor), SP600125 (JNK inhibitor) or DMSO for 2 hrs, then stimulated with HG at 33 mM for 24 hrs. After treatment, total RNA were extracted and the mRNA levels of angiotensin converting enzyme (ACE) (A), transforming growth factor-β1 (TGF-β1) (B) and Renin (E) were analysed by RT-qPCR (normalized to GAPDH gene, *n* = 4); or cell lysates were collected and ACE (C) and TGF-β1 (D) proteins were detected by Western blot analysis. The column figures show the normalized optical density from the data more than three independent experiments. (F) A schematic illustration of the protection of C66 against HG-induced ACE expression and renal damage *via* MAPK inactivation and ACE down-regulation.

In order to validate the possibility that MAPK signalling regulates ACE expression, we pre-treated cells with MAPK-specific inhibitors before HG incubation. As shown in Figure 5, all of the three MAPK inhibitors (PD98059 as ERK inhibitor, SB235035 as p38 inhibitor and SP600125 as JNK inhibitor) exhibited similar inhibitory effects on the transcriptional expression of ACE (Fig. 5A) and TGF-β1 (Fig. 5B). Similar results were observed at the level of protein expression (Fig. 5C and D). Consistent with the *in vivo* result, MAPK inhibitors did not affect HG-induced gene expression of renin (Fig. 5E). These results demonstrate that MAPK signalling could regulate the transcriptional expression of ACE, indicating that MAPKs may affect RAS activity *via* regulation of ACE, rather than renin.

## Discussion

Diabetic nephropathy has become the most common cause of end-stage renal disease. The STZ-induced diabetic mouse model has been widely used to study early diabetic renal changes. In this study, we demonstrate that a novel curcumin analogue, C66, efficiently attenuated diabetic renal injury *via* inhibition of MAPK-mediated ACE expression and RAS activation. Importantly, it is observed that the MAPK signalling pathway may contribute to the development of DN *via* a new ACE-involved mechanism.

Renin-angiotensin system is a major contributor to systemic blood pressure, while recently, it has also been implicated as playing an important role in the development of DN 2. Renin-angiotensin system components, including angiotensinogen, renin, ACE and Ang II, were abnormally increased in STZ-induced diabetic rat models 21,22. The mRNA expression of RAS components and the production of Ang II were also increased in HG-incubated NRK-52E cells 23. More importantly, these results agreed with observations in diabetic patients 2,24. Our present results also show that these RAS elements were all increased in diabetic mouse kidneys (Fig. 2A–D), and that HG dose-and time-dependently stimulated the expression of ACE in renal tubular cells (Fig. 3A–D). The intrarenal influences of RAS signalling are executed by Ang II *via* binding to its specific membrane receptor (AT1), which ultimately leads to renal fibrosis and injury 25,26. Recently, a myriad of experimental studies have suggested that non-haemodynamic mechanisms including attenuation of TGF-β1 expression and fibrosis, also contribute to inhibition of Ang II-induced renal injury and the renoprotective effects of RAS blockade 25,27,28. Previous studies have demonstrated that Ang II could stimulate TGF-β1 expression and fibrosis both *in vitro* and *in vivo* 14,25. High glucose-induced fibrosis accumulation was able to be prevented by the AT1 blocker losartan, indicating that Ang II/AT1 mediate the renal TGF-β1 increase and fibrosis observed in type 1 DM 29. Therefore, TGF-β1 could be regarded as a hallmark sign in RAS-mediated renal fibrosis and injury. The present study also found that the fibrosis was markedly increased in diabetic mouse kidneys (represented by collagen IV staining, Fig. 1D), and that HG incubation increased the expression of TGF-β1 (Fig. 4B and D).

A number of studies in human and experimental animals have shown that blockade of the RAS pathway could protect the kidney from DN 21,30,31. Angiotensin converting enzyme is the key rate-limiting enzyme in Ang II formation and it has been a potential target for the therapy of DN 2. A recent clinical study using 409 patients with DN showed that patients treated with the ACE inhibitor captopril were associated with a 48% reduction in serum creatinine, and a 50% reduction in death 32. However, previous studies on ACE inhibition were mostly performed with small molecule inhibitors which antagonize the enzyme activity of ACE. Studies in both humans and experimental animals have shown significant increases in ACE gene expression in diabetic kidneys 33,34. Although a few agents, including telmisartan and aprotinin, have been reported to attenuate diabetic renal injury accompanied by a decrease in ACE expression in animal kidneys 35,36, it is not very clear whether inhibition of ACE expression could affect the progression of DN. The results shown in Figures 2C and 4 also show that both hyperglycaemia and HG stimulate ACE expression. Administration with C66 negatively regulated hyperglycaemia-or HG-induced ACE expression and subsequent Ang II increase, which was associated with the renoprotective effects of C66 in diabetes. These findings indicated that inhibition of ACE expression could efficiently attenuate DN.

The regulation of ACE gene expression in DN is not well understood. The ACE gene promoter contains a number of consensus transcription factor binding sites, including the early growth responsive gene-1 (Egr-1), specificity protein-1 (SP-1), E26 transformation-specific-1 (ET-1), and activator protein-1 (AP-1) 37,38. Previous studies suggest that the increase in ACE mRNA transcription induced by growth factor, phorbol 12-myristate 13-acetate, and bleomycin was mediated by MAPKs and protein kinase C 12,37,39. However, it is unclear whether MAPKs regulate ACE expression and subsequent Ang II synthesis under HG stimulation. In contrast, studies have demonstrated that MAPKs, as downstream signalling kinases, could be activated by Ang II and mediate a series of Ang II-induced pathophysiological actions 14,15. It has been demonstrated that HG incubation or hyperglycaemia markedly stimulated the phosphorylation of MAP kinases, which may mediate renal inflammation, macrophage infiltration and renal dysfunction 8. Inhibition of MAPK signalling effectively avoided HG-induced renal cell damage and nephropathy 19, suggesting that it may be a potential therapeutic target for DN. Our results shown in Figure 2A reveal the marked activation of MAPKs in diabetic kidney tissues, accompanied by RAS activation (Fig. 2B–E). *In vitro*, a time-course effect of HG on MAPK phosphorylation showed that MAPKs are activated by HG stimulation in a very short time (15 min.), suggesting that MAPKs may be the upstream signalling pathway in the HG-ACE-TGF-β1 cascade (Fig. 3E). MAPK-specific inhibitors were then used to confirm their effects on HG-induced ACE and TGF-β1 expression. Interestingly, all of the three inhibitors at 10 μM could block HG-induced overexpression of both ACE and TGF-β1 in renal tubular cells (Fig. 5A–D), suggesting that MAPKs mediate HG-stimulated ACE expression. Previous studies have demonstrated that the transcriptional factor AP-1 could regulate ACE gene expression 37,38. On the other hand, AP-1 has been widely known as an important downstream mediator of MAPK pathway. Three different types of MAPKs, the ERK, the JNK and p38, contribute to induction of AP-1 activity in response to a diverse array of extracellular stimuli. Especially, it is of considerable interest that each of these types of MAPKs is affecting AP-1 activity through phosphorylation of a different substrate 40. Thus, it is extremely likely that MAPKs up-regulated the expression of ACE gene *via* activating AP-1. Further studies in the future are necessary to establish such notion.

More interestingly, the HG-induced renin expression seems to be MAPKs-independent. MAPK-specific inhibitors showed no inhibitory effect on renin mRNA expression (Fig. 5E). This is also confirmed by the *in vivo* data that show that C66 administration only inhibited ACE expression and the subsequent serum Ang II level, but did not affect the upstream renin and angiotensinogen expression in diabetic kidneys. Thus, it is concluded that MAPKs mediate HG-induced RAS activation *via* inhibition of ACE gene transcription and expression.

C66, a new structural analogue of curcumin developed by our laboratory in the past 5 years, is a promising agent for the treatment of DN. We previously reported that C66 was able to reduce HG-induced inflammation profiles both *in vitro* and *in vivo*, accompanying by its inhibition on JNK phosphorylation, and then prevented renal injury in experimental diabetic rats 19. It is now in the later stage of a pre-clinical study as a new therapeutic candidate for DN (data not shown). In this study, C66 significantly inhibited HG-induced MAPK phosphorylation both *in vitro* (Fig. 4F) and *in vivo* (Fig. 2A). Mitogen-activated protein kinases may be the molecular targets of C66 for its biological functions. The previous mechanistic insights demonstrated the pharmacological pathway of the anti-inflammatory actions of C66 19. Here, we further showed that C66 could affect HG/hyperglycaemia-induced renal RAS activation, including ACE expression and subsequent Ang II secretion, in a MAPK-dependent manner. This new mechanism also contributes to the renoprotective actions of C66 in diabetes. C66 treatment did not decrease the STZ-caused hyperglycaemia. Consistent with the previous data in diabetic rats, it was found that C66 treatment significantly reduced renal collagen accumulation and fibrosis, and attenuated circulating pathological indexes of renal injury in the DM. These pharmacological effects were associated with its MAPK-dependent inhibition against the increase in renal ACE, Ang II, and TGF-β1 (Fig. 2). In addition, the above pharmacodynamic and mechanistic actions of C66 were repeated and confirmed in HG-stimulated renal cells (Fig. 4).

In summary, a schematic illustration for the prevention of C66 from HG-induced RAS activation and renal injury is shown in Figure 5F. Mitogen-activated protein kinases mediated the hyperglycaemia-induced ACE expression in diabetic kidneys, which contributed to the renal Ang II activation and the development of DN. A synthetic curcumin analogue C66, targeting MAPKs, could decrease HG-induced ACE/Ang II/TGF-β1 both *in vitro* and *in vivo,* and attenuated diabetic renal fibrosis and pathological injury in mice, further indicating that C66 is a potential candidate for the treatment of DN. More importantly, this study provides a new concept that the MAPK pathway is involved in HG-induced RAS activation and renal fibrosis *via* regulation of ACE expression in the development of DN, indicating that reduction in ACE expression by MAPKs inhibition seems to be an alternative strategy for the treatment of DN.

## Supporting information

**Figure S1** C66 treatment did not affect the profile of blood glucose, but significantly reduced diabetes-induced plasma creatinine level in the diabetic mice. (A) The profiles of blood glucose level in three groups are shown. (B) Plasma creatinine in diabetic mice was reduced by C66 treatment. **Table S1.** Gene primer sequences for real-time quantitative PCR analysis.
