# Supplementary material for: Inhibition of MAPK-mediated ACE expression by compound C66 prevents STZ-induced diabetic nephropathy
Source: J Cell Mol Med. 2013 Dec 11;18(2):231–41. doi: 10.1111/jcmm.12175 (PMC3930410; doi:10.1111/jcmm.12175)
Supplement: Supplementary file 1 — Figure S1 C66 treatment did not affect the profile of blood glucose, but significantly reduced diabetes-induced plasma creatinine level in the diabetic mice. (A) The profiles of blood glucose level in three groups are shown. (B) Plasma creatinine in diabetic mice was reduced by C66 treatment. Table S1. Gene primer sequences for real-time quantitative PCR analysis. [file jcmm0018-0231-sd1.doc]

**Supporting Information**

**Inhibition of MAPKs-mediated angiotensin-converting enzyme expression by compound C66 prevents STZ-induced diabetic nephropathy**

Yong Pan^1,2,*^, Yusheng Sun^3^,*, Zhe Wang^1^, Qilu Fang^1^, Chao Tong^1^, Kesong Peng^1^, Lu Cai^2^, Yunjie Zhao^1, #^, and Guang Liang^1, #^

*^1^ Chemical Biology Research Center, School of Pharmaceutical Scieneces, Wenzhou Medical University, Wenzhou, Zhejiang, 325035, P. R. China.*

*^2^ Chinese-American Research Institute for Diabetic Complications, School of Pharmaceutical Scieneces, Wenzhou Medical University, Wenzhou, Zhejiang, 325035, P. R. China.*

*^3^ Department of General Surgery, The First Affilliated Hospital of Wenzhou Medical University, Wenzhou, Zhejiang, 325000, P.R. China*

**C66 treatment did not affect the profile of blood glucose, but significantly reduced diabetes-induced plasma creatinine level in the diabetic mice**

**Materials and Methods**

Protocols for animal studies were approved by the Wenzhou Medical College Animal Policy and Welfare Committee (Approved documents: 2009/APWC/0031). Male C57BL/6 mice, weighing 18-22g at 8 weeks of age, were obtained from the Animal Center of Wenzhou Medical College (Wenzhou, China). Animals were housed at 22 °C with a 12:12 h light/dark cycle and water and a standard mouse diet were consumed. To induce type 1 diabetes, mice were treated with a single intraperitoneal injection of streptozotocin (STZ; 150 mg/kg in citrate buffer, pH=4.5), while the control animals were received the same volume of citrate buffer. The blood glucose level was monitored on days 3 and 7 after the STZ injection using a glucometer. Seven days after STZ injection, mice with fasting-blood glucose >12 mmol/L were considered diabetic, and then randomly divided into two groups: diabetic mice (DM, n=7), and C66-treated diabetic mice (DM+C66, n=7). In the DM+C66 group, mice were orally administrated with C66 at 5 mg/kg once every 2 days. The DM group and age-matched control group (n=7) received 1% CMC-Na solution alone according to the same schedule. Blood glucose levels were recorded on days 7, 17, 27, 47, 57, and 67 after C66 administration. On day 67, animals were sacrificed under ether anesthesia. The blood was also collected at the time of death and the levels of serum creatinine were determined by an automatic biochemical analyzer (Olympus AU5400, Olympus Corporation, Japan) according to the manufacturer's instructions.

**Results**

To validate the beneficial functions of C66 *in vivo*, a model of STZ-induced diabetic mice was used. Seven days after STZ injection, all mice were developed overt diabetes. The mice in DM+C66 group were orally treated with C66 at 5 mg/kg/day for 10 weeks. Figure S1A showed that there was no significant difference in blood glucose levels between the DM-control and DM+C66-treated group, indicating that C66 treatment did not affect blood glucose level in the diabetic mice. Sixty seven days after STZ induction, mice were sacrificed and the kidneys were analyzed. Serum creatinine is one of the hallmarks of renal injury. Figure S1B showed that 67 days after STZ induction, the mean serum creatinine level of the DM group was significantly higher than those of the control (*p*<0.01) and the C66-treated diabetic mice (*p*<0.01).


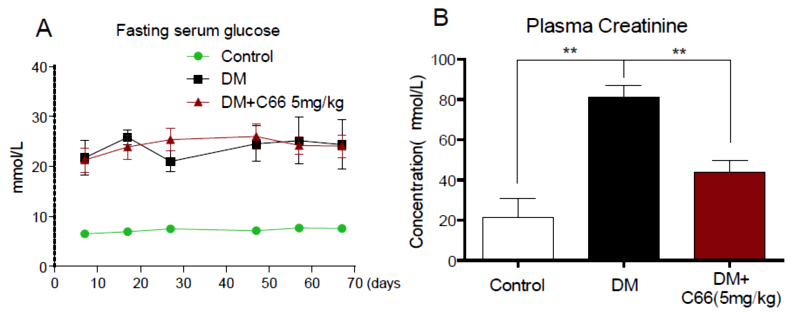


**Figure S1.** C66 treatment did not affect the profile of blood glucose, but significantly reduced diabetes-induced plasma creatinine level in the diabetic mice. A. The profiles of blood glucose level in three groups are shown. B. Plasma creatinine in diabetic mice was reduced by C66 treatment.

**Table S1.** Gene primer sequences for real-time quantitative PCR analysis.

| **Source** | **Gene** | **Sequence 5’-3’ ( forward)** | **Sequence 5’-3’ ( reverse)** |
| --- | --- | --- | --- |
| Rat | ACE | CACCGGCAAGGTCTGCTT | CTTGGCATAGTTTCGTGAGGAA |
|  | TGF-β1 | GCAACAACGCAATCTATGAC | CCTGTATTCCGTCTCCTT |
|  | renin | ACCAGGGCAACTTTCACTACGT | ACCCCCTTCATGGTGATCTG |
|  | GAPDH | AAGTCCCTCACCCTCCCAAAAG | AAGCAATGCTGTCACCTTCCC |
| Mouse | angiotensinogen | TATCCACTGACCCAGTTCTT | AAGTGAACGTAGGTGTTGAAA |
|  | renin | TTGTTGCTCTGGAGTCCTTGC | CAGGATTTCCCGGACAGAAGG |
|  | TGF-β1 | GCAACAACGCAATCTATGAC | CCTGTATTCCGTCTCCTT |
|  | ACE | GCAACAACGCAATCTATGAC | CCTGTATTCCGTCTCCTT |
|  | β-actin | TGGAATCCTGTGGCATCCATGAAAC | TAAAACGCAGCTCAGTAACAGTCCG |
